# Exposure measurement error in air pollution studies: A framework for assessing shared, multiplicative measurement error in ensemble learning estimates of nitrogen oxides

**DOI:** 10.1016/j.envint.2018.12.025

**Published:** 2019-02-01

**Authors:** Mariam S. Girguis, Lianfa Li, Fred Lurmann, Jun Wu, Robert Urman, Edward Rappaport, Carrie Breton, Frank Gilliland, Daniel Stram, Rima Habre

**Affiliations:** a Division of Environmental Health, Department of Preventive Medicine, Keck School of Medicine, University of Southern California, Los Angeles, CA, USA; b Sonoma Technology, Inc., Petaluma, CA, USA; c Department of Public Health, College of Health Sciences, University of California, Irvine, CA, USA; d Division of Biostatistics, Department of Preventive Medicine, Keck School of Medicine, University of Southern California, Los Angeles, CA, USA

## Abstract

**Background:**

Increasingly ensemble learning-based spatiotemporal models are being used to estimate residential air pollution exposures in epidemiological studies. While these machine learning models typically have improved performance, they suffer from exposure measurement error that is inherent in all models. Our objective is to develop a framework to formally assess shared, multiplicative measurement error (SMME) in our previously published three-stage, ensemble learning-based nitrogen oxides (NO_x_) model to identify its spatial and temporal patterns and predictors.

**Methods:**

By treating the ensembles as an external dosimetry system, we quantified shared and unshared, multiplicative and additive (SUMA) measurement error components in our exposure model. We used generalized additive models (GAMs) with a smooth term for location to identify geographic locations with significantly elevated SMME and explain their spatial and temporal determinants.

**Results:**

We found evidence of significant shared and unshared multiplicative error (p < 0.0001) in our ensemble-learning based spatiotemporal NO_x_ model predictions. Unshared multiplicative error was 26 times larger than SMME. We observed significant geographic (p < 0.0001) and temporal variation in SMME with the majority (43%) of predictions with elevated SMME occurring in the earliest time-period (1992–2000). Densely populated urban prediction regions with complex air pollution sources generally exhibited highest odds of elevated SMME.

**Conclusions:**

We developed a novel statistical framework to formally evaluate the magnitude and drivers of SMME in ensemble learning-based exposure models. Our framework can be used to inform building future improved exposure models.

## 1. Introduction

Exposure to traffic-related air pollution (TRAP) has repeatedly been associated with mortality and adverse health outcomes, including respiratory illnesses and cardiovascular disease, in large epidemiological cohort studies of children and adults (Zhang et al., 2002; Andersen et al., 2008; Gehring et al., 2010; Esposito et al., 2014; Ryan et al., 2005; Nordling et al., 2008; Chen et al., 2015; Rancière et al., 2017; Pollution HEIPotHEoT-RA, 2010). Nitrogen oxides (NO_X_), which are byproducts of fuel combustion, are one of the most commonly used measures of TRAP in epidemiological studies. NO_X_ are also precursor gases involved in the secondary formation of ozone and particulate matter - air pollutants also implicated in adversely affecting health (Rancière et al., 2017; Goldsmith and Kobzik, 1999; Khreis et al., 2017; Schwela, 2000). NO_x_'s highly reactive nature results in dynamic variability in space and time (Apte et al., 2017), limiting the utility of traditional exposure assessment methods that rely solely on interpolation from sparse central site monitoring data or land use regression techniques, which typically suffer from poor spatial and temporal resolution, respectively (Sheppard et al., 2012). Similarly, crude spatially-derived surrogates of TRAP such as distance to roads or traffic density within buffers often covary in space with potential confounders such as socioeconomic status, access to health care, or other environmental and psychosocial exposures (Pollution HEIPotHEoT-RA, 2010). Therefore, sophisticated spatiotemporal exposure models that incorporate machine learning techniques are increasingly being developed to more accurately predict residential TRAP exposures (and other complex spatially and temporally varying exposures) (Li et al., 2017; Russo and Soares, 2014; Di et al., 2016), given that ‘gold standard’ personal monitoring to capture ‘true exposure’ is often not feasible in large cohort studies. However, spatial and temporal uncertainties inherent in these exposure models result in a complex correlation structure which leads to error in exposure predictions, referred to as exposure measurement error. These errors can be categorized as independent (unshared) or dependent (shared).

Depending on its structure, exposure measurement error can result in decreased precision and/or biased epidemiological inference (Zeger, 2001; Zeger et al., 2000; Carroll, 1998). Classical error, *W* = *T* + *E*, where *W* is the measured (surrogate) exposure, *T* is the true exposure, and *E* is random error, assumes that *E* has a mean equal to zero and is independent of *T*, while Berkson error, *T* = *W* + *E*, assumes that *E* has a mean equal to zero and is independent of *W* (as opposed to *T* in the classical error scenario). Further, exposure errors can take an additive (as demonstrated above) or multiplicative structure (additive error on the log scale) (Heid et al., 2004). A multiplicative error structure, common in air pollution exposure measurements, can alter exposure-response shapes (over and/or under estimation) and applies when the error is proportional to the true exposure (Lyles and Kupper, 1997).

Shared error can occur because of shared uncertainties in exposure predictions due to spatial and/or temporal misalignment of exposure predictors. For example, temperature is often included in spatio-temporal NO_x_ exposure models. But temperature may not be available at the same spatial resolution as predictions, resulting in NO_x_ measurement error due to inaccuracies associated with readings of temperature from a single instrument applied to all prediction points in a defined spatiotemporal grid. Shared Berkson error occurs if all or groups of prediction points within the defined spatiotemporal grid are misrepresented in the same way. Shared classical measurement error can occur when the average temperature across space or time is not the true average of all prediction points included in the defined spatio-temporal grid. Both scenarios violate the independence assumption of exposure (true and measured, respectively) and error. Shared error can be both classical-like or Berkson-like (Mallick et al., 2002) and results from spatial and/or temporal covariance between exposure predictions.

Recently, our group developed a sophisticated three-stage spatio-temporal modeling framework with ensemble learning and constrained optimization to model NO_X_ concentrations in southern California for use in epidemiological studies of children's health (Li et al., 2017). In addition to a typical single stage model where a spatiotemporal mixed-effects model is fit, a second stage with ensemble learning using bootstrap aggregation is employed. This machine learning technique combines the output from hundreds of individual learners in a weighted fashion and results in decreased variance in the predictions (higher precision). Constrained optimization is then applied in a third stage to adjust predictions to better reflect reality based on known physical and chemical constraints, improving overall accuracy and decreasing bias in the NO_x_ exposure estimates. We have already demonstrated the improved performance of our modeling framework in predicting NO_x_ exposures in southern California (R^2^: 0.86, RMSE: 13.4) (Li et al., 2017); however, we have not yet assessed the uncertainties inherent in these exposure predictions.

In the current work, we aim to formally evaluate the magnitude of shared and unshared, multiplicative and additive (SUMA) measurement error components in our Li et al. (2017) southern CA NO_x_ model (1992–2013) predictions using a statistical dosimetry framework developed by Stram and Kopecky (2003). We expand by providing a framework to explain the geographic and temporal determinants of the shared multiplicative measurement error (SMME) component.

## 2. Methods

**Fig. 1.**
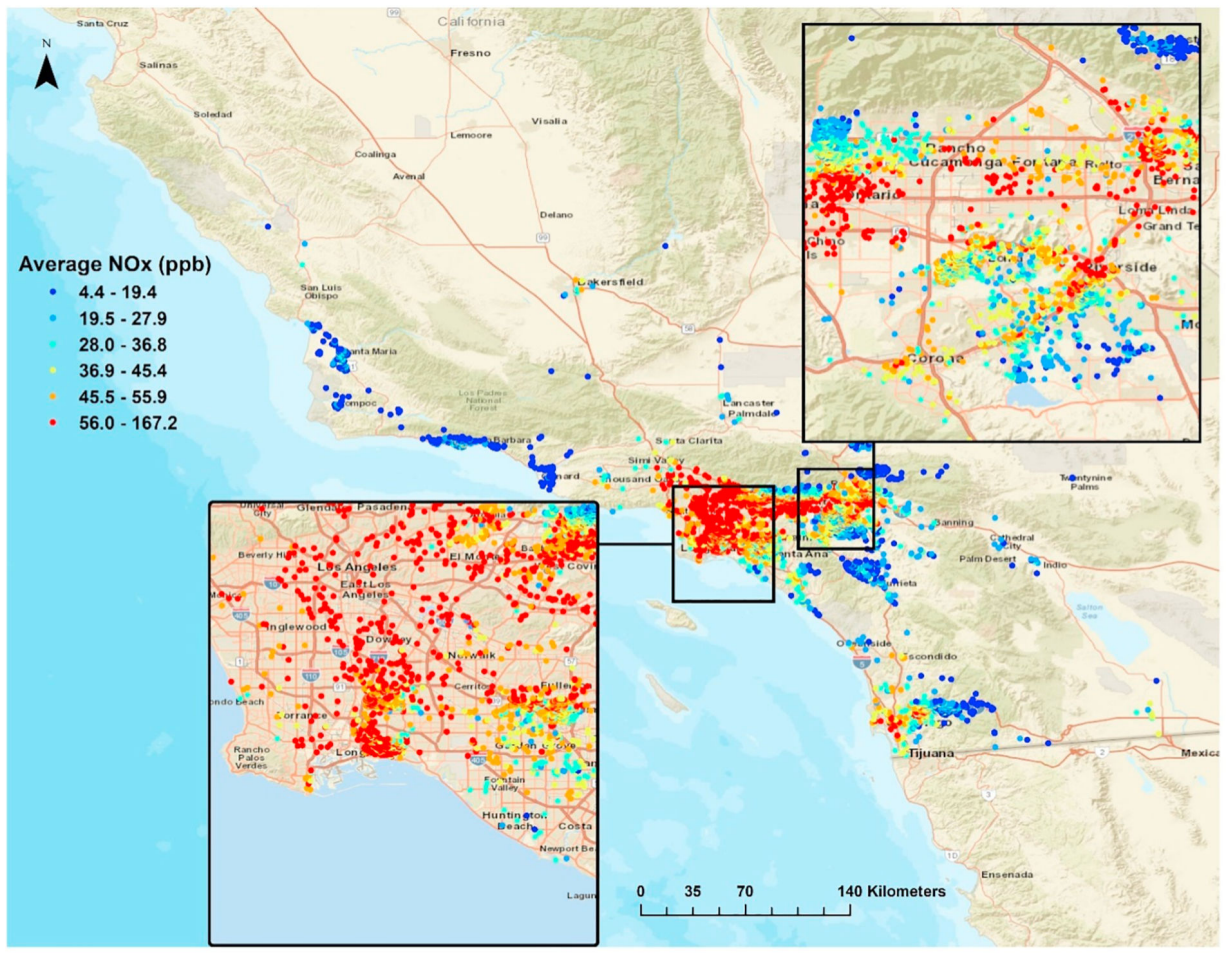
Average NO_x_ (ppb) for southern California Children's Health Study (CHS) residential locations, 1992–2012. Average NO_x_ using stage 3 of the Li et al. (2017) model which uses the averaged stage 2 NO_x_ estimates and constrained optimization to re-predict exposure based on physical constraints meant to mimic known or observed real-life behavior of NO_x_. Average NO_x_ for each unique CHS location displayed using quantiles (6).

This investigation will use NO_x_ exposure predictions for the most recent cohort (E) of the southern California Children's Health Study (CHS) (Chen et al., 2015; Peters et al., 1999) which started enrolling participants in 2002 with prenatal periods starting in 1992. Information from longitudinal address confirmation, residential history questionnaires and birth certificates was used to assemble lifetime residential histories for these participants and assign biweekly NO_x_ exposure based on our model (Li et al., 2017). TRAP exposures were assigned to CHS participants across their lifetime using the novel machine learning spatiotemporal NO_x_ model described in more detail in Li et al. (2017) to estimate residential NO_x_ exposures at high spatio-temporal resolution (Li et al., 2017). Briefly, the model uses a flexible hierarchical framework with spatiotemporally-referenced covariates and measurement data from both long-term routine monitoring stations with high temporal resolution and short-term, sporadic measurement campaigns with high spatial resolution. Temporal basis functions are fit on the long-term monitoring data using singular value decomposition to capture seasonality and longer term temporal variation (Szpiro et al., 2010). Stage 1 of the model uses temporal parameters, long term mean concentrations, and local spatial predictors including line dispersion CALINE4 NO_x_ estimates (Benson, 1984), traffic density, distance to major roads, population density, and meteorological parameters (wind speed and minimum temperature) to model NO_x_ concentrations. Spatial effects were specified both as random effects based on 500 m aggregate distance Thiessen polygons and nonparametric additive terms. Stage 2 iteratively samples 90% of the predictors used in stage 1 and a random subset of 63% of the observations to test against the remaining 37% of the data set in each ensemble, obtaining 120 individual mixed-effect models (referred to as ensembles) that produce biweekly predictions. The estimates from the 120 ensembles are subsequently averaged (weighted by model performance) to provide optimal NO_x_ predictions across the distribution of the data that are robust against investigator bias through forced covariate inclusion and inflated variance of predictions (referred to as stage 2 NO_x_ predictions). Stage 3 of the model uses the averaged stage 2 NO_x_ estimates and constrains the parameter estimates of the temporal basis functions to re-predict exposure based on physical constraints meant to mimic known or observed real-life behavior of NO_x_ (e.g. decreasing temporal trend of NO_x_ over study years, NO_2_ output less than NO_x_ output, higher cool season concentrations compared to warm season, etc.). This third stage is known as constrained optimization and its output is referred to as stage 3 NO_x_ predictions (Li et al., 2017; Russo and Soares, 2014) (Fig. 1).

### 2.1. Using stage 2 ensembles as a dosimetry system

The second stage output of the 120 ensembles allows for a unique opportunity to evaluate SUMA exposure measurement error. To quantify the various forms of measurement error, we treated the 120 ensemble predictions as 120 realizations generated from an external dosimetry system. An external dosimetry system is typically used in radiation exposure literature to reconstruct distributions of radiation dose through calculation and assessment of radiation exposure based on knowledge of the physical processes and sources of irradiation (Boyd, 2009). In a similar fashion to radiation dose, NO_x_ residential exposure estimates were reconstructed. We assume the 120 NO_x_ ensembles are sampled from the distribution of true exposure. Each ensemble includes biweekly NO_x_ exposure predictions for all CHS participants across their life course. Using these 120 ensembles, each SUMA component of exposure measurement error is quantified. As the ensembles are presumed to be coming from a distribution of true exposure given the known exposure determinants, adjustment for measurement error is based on a Berskon model.

### 2.2. Statistical analysis

#### 2.2.1. Quantifying SUMA error components

All references to a NO_x_ exposure prediction from here onward are for a two-week estimate for a given subject and location (denoted by “i”), unless otherwise noted. The SUMA model for shared and unshared Berkson error is written as follows:

(1) $$X_{i}=\epsilon_{SM}\epsilon_{Mi}Z_{i}+\epsilon_{SA}+\epsilon_{Ai}$$

Here *X_i_* is the true exposure for the estimate of interest, *Z_i_* is the estimated exposure (a weighted mean of the ensembles). *ϵ_SM_* and *ϵ_Mi_* are the shared and unshared multiplicative errors with mean equal to 1 and variances *σ_SM_*^2^ and *σ_M_*^2^ respectively, and *ϵ_SA_* and *ϵ_Ai_* are the shared and unshared additive errors, with mean equal to 0 and variances *σ_SA_*^2^ and *σ_A_*^2^ respectively.

Our focus in the remainder of the manuscript is primarily on the variance of the shared multiplicative error component (*σ_SM_*^2^) because this variance term is what primarily affects the behavior of variance estimates and confidence intervals for the slope term in a standard regression analysis used in an epidemiological investigation of an exposure estimate *W* on outcome *D*.

Assuming that each of the ensembles are samples from the true distribution of exposure (Eq. (1)) then Stram and Kopecky (2003) propose estimating the four variance terms *σ_SM_*^2^, *σ_M_*^2^, *σ_SA_*^2^, and *σ_A_*^2^ as follows.

#### 2.2.2. Shared measurement error

For each pair of NO_x_ predictions, *i* and *j*, we calculated the covariance of the realized values of *X_i_* and *X_j_* over the 120 ensembles and called this covariance term *C_ij_*. At the same time, we calculated the *Z_i_* and *Z_j_* values as the mean of the realized values of *X_i_* and *X_j_* (stage 2 exposure predictions as explained earlier). Next, we performed simple ordinary least squares (OLS) regression of *C_ij_* on the product *Z_i_Z_j_* to fit the model

(2) $$C_{ij}=a_{0}+a_{1}Z_{i}Z_{j}+\varepsilon_{ij}$$

Stram and Kopecky note that the intercept term, *a*_0_ in this regression corresponds to $\hat{\sigma_{\mathrm{SA}}^{2}}$, which is an estimate of *σ_SA_*^2^, while the slope term (*a*_1_) corresponds to $\hat{\sigma_{\mathrm{SM}}^{2}}$ or the estimate of *<_SM_*^2^.

#### 2.2.3. Unshared measurement error

Similarly, we calculated the variance of each *X_i_* across ensembles, *V_i_*, which is shown to equal the following (Stram and Kopecky, 2003):

(3) $$Z_{i}^{2}[(\sigma_{SM}^{2}+1)(\sigma_{M}^{2}+1)-1]+\sigma_{SA}^{2}+\sigma_{A}^{2}$$

We then used simple OLS regression of *V_i_* on *Z_i_*^2^, which allows for the estimation of *σ_SA_*^2^ + *σ_A_*^2^ (as the intercept term) and [(*σ_SM_*^2^ + 1) (*σ_M_*^2^ + 1) – 1] (as the slope term) to solve for $\hat{\sigma_{M}^{2}}$ an estimate of *σ_M_*^2^ and $\hat{\sigma_{A}^{2}}$, an estimate of *σ_A_*^2^.

Due to the intensity and duration of calculation, a random subset of 2500 NO_x_ predictions were selected for SUMA error quantification. To confirm the sample of 2500 NO_x_ predictions were representative of our model and there was no bias introduced by the random sampling, 10 additional random samples were selected (for a total of 11) and the above analysis was repeated to confirm robustness of results. We further compared the distributions of time and geographic characteristics of the sampled predictions to those of the full NOx exposure predictions.

### 2.3. Spatial and temporal determinants of ‘high’ shared multiplicative measurement error (SMME)

#### 2.3.1. Defining ‘high’ SMME for each prediction

**Fig. 2.**
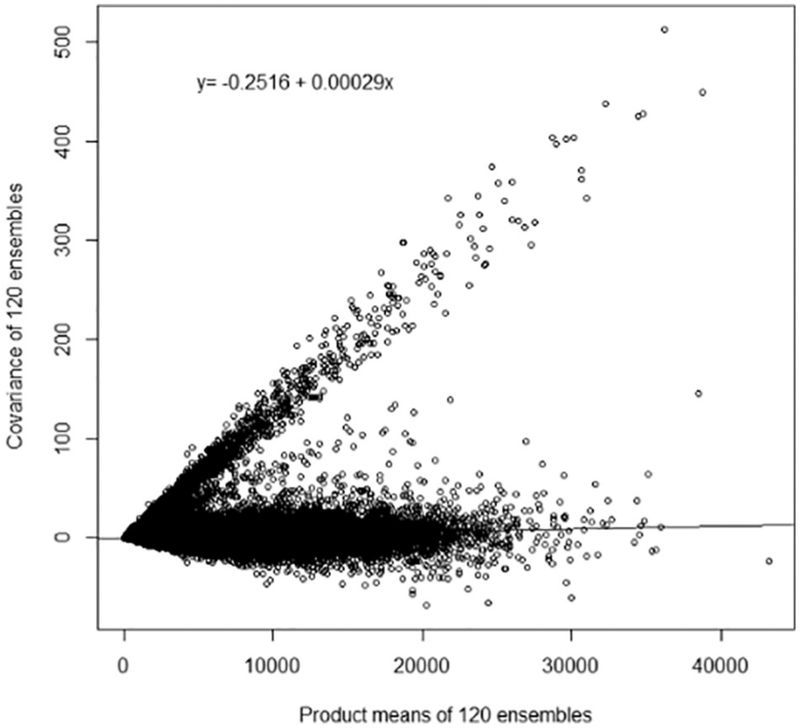
Scatter plot of covariance by product means to visualize shared exposure measurement error. The covariance and product of means of each pair of predictions are used to demonstrate shared error. The intercept of the ordinary least squares regression line to fit the data is −0.2516 with a slope of 0.000029. The negative intercept indicates there is no evidence of additive shared error and the significant slope (p < 0.0001) indicates significant multiplicative shared error.

For each prediction *i*, we calculated the “mean covariance” as the mean *C_ij_* over all other predictions *j* of (*Z_i_* – *E*(*Z*))(*Z_j_* – *E*(*Z*)). We expect that a prediction that consistently covaries with other predictions will yield an elevated average covariance, indicating increased shared uncertainties, while a prediction that covaries with few other predictions will yield a low average covariance, representing decreased shared uncertainties within the prediction. Based on observed bimodality in the distribution of the mean covariances, each prediction was assigned a dichotomized value of “high” (upper 20th percentile of average covariances for each prediction) or “low” (below the 80th percentile of average covariance for each prediction) SMME. Dichotomization at the 80th percentile was used as the cut off based on a visual inspection of the plotted covariance and product means (Fig. 2).

Descriptive summaries of the exposure model inputs and additional spatiotemporal parameters were summarized and compared for the low versus high SMME groups to describe factors significantly different between locations with low versus high SMME.

#### 2.3.2. Temporal analysis

To assess temporal trends in SMME, similar analyses were performed only stratified by time, defined as tertiles of calendar year as follows: 1992–2000, 2001–2004, and 2005–2012. For each time-period, a (new) random sample of 2500 NO_x_ predictions was selected. SMME was calculated and compared for each time-period.

#### 2.3.3. Spatial analysis

Generalized additive models (GAMs) with a smooth term for location were used to assess spatial variability of SMME (Girguis et al., 2016). The following GAM was fit to model the odds of high SMME (compared to low as the reference group):

(4) $$\text{logit}[p(x_{1},y_{1})]=s(x_{1},y_{1})+\gamma^{\prime }$$

where logit[*p*(*x*_1_, *y*_1_)] is the log-odds of high SMME at location (*x*_1_, *y*_1_), *s* (*x*_1_, *y*_1_) is a bivariate locally weighted scatterplot smoothing (loess) function at location (*x*_1_, *y*_1_) capturing the contribution of geographic location and *γ*‱ is a vector of spatial and/or temporal parameters explored in the model. Odds of high SMME were predicted across a grid of evenly spaced points constrained by the geographical extent of CHS lifetime residential locations in Southern California (as NO_x_ predictions were only made in Southern California). A confidence band with an alpha = 5 × 10^−7^ (determined by false discovery rate correction) for each grid point was calculated to identify areas of statistically increased or decreased SMME. An unadjusted GAM with only a term for location was used to determine the existence of spatial variability of high SMME. GAMs were then run iteratively, adding a single predictor at a time, to assess the importance of each predictor in explaining the spatial variability of high SMME. Predictors were selected to be included in the final model if a) they significantly altered the spatial patterns of SMME or b) they influenced the range (minimum and maximum odds ratio) of SMME unexplained after their inclusion.

To determine each potential predictor's influence on spatial patterns of SMME the following predictors considered for inclusion in the GAM: NO_x_ measures (including spatiotemporal predictions and ambient monitoring station measures), traffic measures (including traffic density, distance to nearest road by class (FCC1 through FCC4 class roads defined as freeways, arterial roads, collector distributor roads, and local roads, respectively), meteorological measures (including minimum temperature and wind speed), time (categorized and continuous), and other geographic variables (including distance to shore and population density) to determine each potential predictor's influence on the initial spatial patterns of SMME. See Table A2 for a full list of variables and descriptions. To determine the predictors, influence on spatial patterns of SMME, we visually examined patterns to determine if (1) the geographic locations with statistically significant SMME shifted or changed and (2) if the pattern of SMME risk changed and (3) if the range (max odds ratio and minimum odds ratio across space) of SMME risk across the geographic location changed.

## 3. Results

Characteristics of predicted NO_x_ exposures and key spatiotemporal model predictors for the complete CHS cohort E lifetime residential histories and a random sample of 2500 points are summarized for comparison in Table 1. The distribution of geographical and temporal characteristics between the random sample and the entire dataset was similar confirming the representativeness of the random sample. For all CHS prediction points and the random sample, approximately 85% were located further than 300 m away from major roadways (FCC1).

**Fig. 3.**
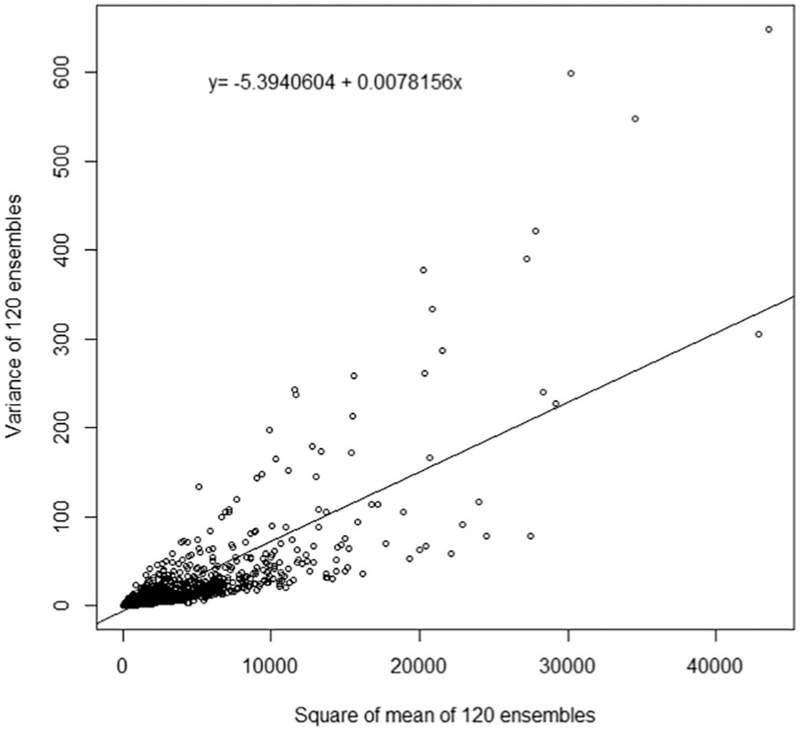
Scatter plot of prediction variance by square of mean to visualize unshared exposure measurement error. The variance and square of mean for each prediction across 120 ensembles are used to demonstrate unshared error. The intercept of the ordinary least squares regression line to fit the data is −5.39 with a slope of 0.0078. The negative intercept indicates there is no evidence of additive unshared error and the significant slope (p < 0.0001) indicates significant multiplicative unshared error.

To quantify SUMA error, we calculated the covariance, product means, variance and square of means from the random sample of exposure predictions. The distributions are shown in Table 2. Quantified SUMA error components as determined by OLS regression are displayed in Table 3. The slope of the regressed covariance on the product mean is statistically significant (p < 0.00001) indicating a SMME value of 0.00029. The intercept, or shared additive error value, is less than zero (−0.2516) indicating no evidence of shared additive error. Similarly, for the unshared error analysis (OLS regression of the variance on the square of means), the intercept is < 0, indicating no evidence of unshared additive error. Although the additive error components (variances) are estimated to be negative, it is clear from Figs. 2 and 3 that the discrepancy between the nominal value of the additive variances and zero is very small. After setting the additive error values ($\sigma_{A}^{2}$ and $\sigma_{\mathrm{SA}}^{2}$) to zero, and solving Eq. (2), unshared multiplicative error is calculated as 0.00751. Comparatively, the unshared multiplicative component is approximately 26 times larger than the shared multiplicative component.

The plot of the covariances and the product means (Fig. 2) reveals the presence of two distinct SMME groups: predictions without shared additive and multiplicative error (intercept and slope around zero) and predictions with highly covarying exposure predictions across replications that display evidence of SMME.

**Fig. 4.**
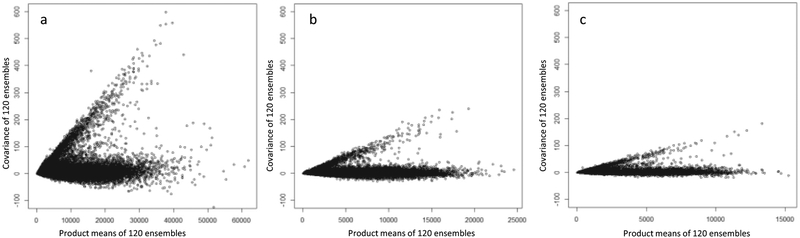
Time stratified visualization of shared error: scatter plot of covariance by product means within random samples from a) 1992–2000, b) 2001–2004, and c) 2005–2012 NO_x_ exposure predictions. Figures include a random subset of 2,500 predictions sampled for each time period stratum.

To quantify SMME and examine how it changes over time, a time stratified analysis was conducted (Table 4). A decreasing trend was observed with the largest SMME found in the earliest time-period, 1992–2000 ($\sigma_{\text{SM}1992-2000}^{2}=0.00036$), and less SMME observed in the subsequent time periods 2001–2004 ($\sigma_{\text{SM}2001-2004}^{2}=0.00015$) and 2005–2012 ($\sigma_{\text{SM}2005-2012}^{2}=0.00014$) (Table 4). Although the magnitude of error decreased across time periods, two distinct SMME groups were consistently observed across the time periods (Fig. 4).

**Fig. 5.**
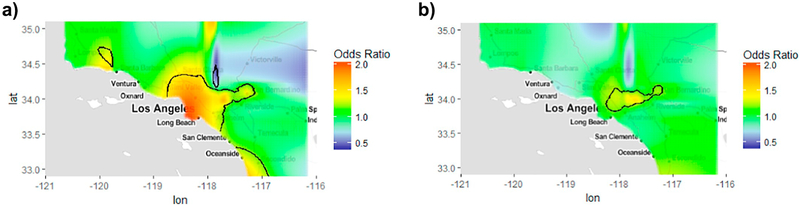
Spatial pattern of the odds of high Shared Multiplicative Exposure Measurement Error (SMME) in Spatiotemporal NO_x_ Predictions for the full southern California Children's Health Study (CHS) Cohort E residential histories in the a) Unadjusted, crude and b) Fully adjusted model. High SMME risk is determined based on the cut-off of the top 80th percentile of average covariance distribution at each unique prediction location. Odds of SMME is adjusted for population density, traffic density, CALINE4 Non-freeway NOx, distance to airport, and prediction year in the fully adjusted model. Statistically significant geographic areas of increased or decreased risk of SMME are indicated using black contour lines.

Spatial analyses using the unadjusted GAM (with only the smooth term for location) showed significant associations between geographic location and covariance distributions (p < 0.0001). Maps indicate the odds of high average covariance which represents high SMME (compared to low, classified based on the 80th percentile of the distribution) ranged from 0.34 up to 2.07 across the entire CHS study area. Areas with statistically significant elevated (hot) or reduced (cold) odds of high SMME are indicated with black contour lines in Fig. 5 (color indicates predicted odds of high SMME specific to that location). The largest risk of high SMME is observed along the southern California coastline.

Geographical and temporal variables were iteratively added to the model to explain the spatial variability observed. The final model included predictors that altered spatial patterns or changed the range of the odds ratios by 10% or more. The final model that best explained the spatial variability in the odds of high SMME included population density, traffic density, CALINE4 Non-Freeway NO_x_, calendar year (categorized into tertiles) and distance to nearest major airport (defined as top 5 class 1 airports in the study region). The Odds Ratio (OR) range decreased (0.50–1.56) and a majority of the spatial variability in SMME risk was explained by the included predictors (Fig. 5b). Few locations remained significantly elevated and were not fully explained. Adjusted GAM results are shown in Table 5 for an interquartile range increase of each predictor. Distance to major airport was the strongest predictor of SMME with predictions located between 0 and 15 km away from a major airport displaying a 1.15 odds (95% Confidence Interval (CI): 1.10, 1.23) of SMME compared to predictions located further than 15 km from major airports. NO_x_ predictions in years following 2000 had decreased odds of high SMME compared to predictions between 1992 and 2000 (OR_2001–2004_: 0.97; 95% 0:0.93, 1.00 and OR_2005–2012_: 0.90; 95% CI:0.87, 0.94) with the lowest odds in later years. Locations with increased traffic density within a 300 m buffer (OR: 1.11; 95% CI:1.09, 1.14), higher population density (OR: 1.03; 95% CI:1.01, 1.04), or higher Non-Freeway CALINE4 NO_x_ (OR: 1.06; 95% CI:1.04, 1.08) also displayed a statistically significantly elevated odds of high SMME.

Although predictions located in the city of Long Beach only make up 6% of the random CHS sample, the largest proportion (23%) of high covariance exposure predictions were found in the city of Long Beach, followed by Anaheim, Riverside, and San Bernardino (8% each) (Table A1). This pattern was consistent across all 10 repeated (for a total of 11) random sample evaluations. Therefore, to separately evaluate the patterns in and predictors of SMME in the city of Long Beach, a random sample of 2500 exposure predictions was re-sampled for predictions within Long Beach. After calculating SUMA components using this Long Beach subsample, we found an SMME value of 0.0021 (seven times larger in magnitude than SMME value calculated for the entire CHS cohort). Exposure model inputs and other predictors related to NO_x_ were compared across “high” (defined as predictions with an average covariance in the upper 20% of Long Beach covariance distributions) and “low” SMME predictions (predictions with an average covariance in the 0–80% of Long Beach covariance distributions) to identify potentially different characteristics (Table 6). High SMME predictions had elevated ambient NO_x_ levels as determined from regional monitoring stations and stage 2 NO_x_ prediction model output. Interestingly, high SMME predictions had higher CALINE4 non-freeway NO_x_ but lower CALINE4 freeway NO_x_ compared to low SMME predictions. Compared to low SMME predictions, high SMME predictions were characterized by the following: higher population density, closer to FCC2 and FCC3 roads but further away from FCC1 and FCC4, closer to the shoreline, greater Heavy Duty Vehicle (HDV) fraction on nearby FCC1 and FCC2 roads, lower average temperatures and slightly higher average wind speeds. There was no difference in elevation across the high and low SMME predictions.

By examining temporal trends in SMME in Long Beach (Table 7), we found that the greatest proportion of NO_x_ predictions with high SMME were observed in the cooler months of winter (39.5%) and fall (35.8%) and the majority of low SMME predictions were observed in the spring (27.5%) and summer (28.9%). Similarly to results using the entire CHS, the highest proportion (43.4%) of high SMME predictions in Long Beach were observed in the earliest time period of 1992–2000.

**Fig. 6.**
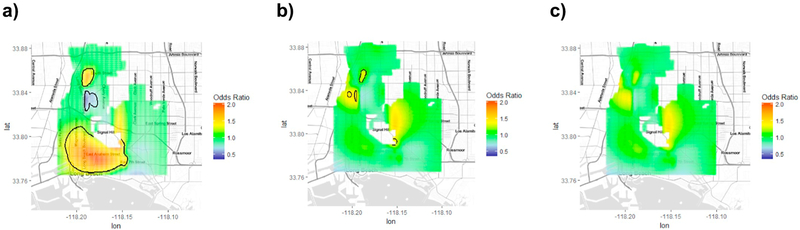
Spatial pattern of the odds of high Shared Multiplicative Exposure Measurement Error (SMME) in spatiotemporal NO_x_ predictions for a random sample of 2500 predictions from the city of Long Beach, CA (a) unadjusted, (b) after spatial (c) and temporal adjustments. High SMME is defined with a cut-off based on the top 80th percentile of average covariance distribution in Long Beach at each unique location. Confounders of shared multiplicative exposure measurement error risk adjusted for in the model included population density, CALINE4 Non-freeway NOx, and Traffic Density on FCC2 Roads. Statistically significant geographic areas of increased or decreased risk of SMME are indicated using black contour lines.

The spatial pattern analysis of Long Beach only using GAMs showed significant associations between geographic location and the odds of high SMME (p < 0.0001). Maps indicate that NO_x_ predictions with elevated odds of high SMME were located in specific regions in southwestern and north Long Beach (Fig. 6). Spatial predictors that best explained the geographic variability in the odds of high SMME in Long Beach included CALINE4 Non-Freeway NO_x_, population density, and traffic density on FCC2 roads (Table A3). After adjusting for these predictors, odds of high SMME in southwestern Long Beach locations were no longer elevated and fewer locations in north Long Beach remained significantly elevated. Geographic variations were only fully explained after including prediction year into the model, reducing the range of the ORs from 0.49–2.03 to 0.67–1.51. Locations with elevated odds of high SMME remained, but these were not statistically significant (Fig. 6).

## 4. Discussion

We recently developed a three-stage NO_x_ spatiotemporal modeling framework to predict exposures at high spatial and temporal resolutions for use in CHS epidemiological analyses. The use of ensemble learning to reduce the variance and minimize bias of exposure predictions in this model is expected to minimize overall exposure measurement error; however, as with all exposure models, it cannot be fully eliminated. Using the Stram and Kopecky (2003) framework, we quantified the SUMA error components in the Li et al. (2017) model predictions. Given that our random sample represents the entire data set, we found evidence of both shared and unshared multiplicative error but no evidence of shared or unshared additive error. The most influential predictors of the odds of high SMME were year of exposure prediction (earlier years had higher error), distance to nearest major airport, and non-freeway NO_x_ concentrations. Overall, we found that unshared multiplicative error was greater in magnitude than SMME when evaluating the full geographical extent of CHS prediction points, but further analysis identified specific geographic regions with relatively high shared multiplicative error. The city of Long Beach, CA, consistently had the highest proportion of NO_x_ predictions with high SMME over several repeated random draws of the data.

We found spatial and temporal patterns in the distribution of SMME in this work. We observed significantly greater SMME in the earliest years (1992–2000) compared to later years (> 2001). This decreasing temporal pattern in the uncertainties is common in retrospective exposure reconstructions (Hoffmann et al., 2018) and may be the result of measurement methods improving or changing over time (for example, a shift from using Palmes tubes to Ogawa badges for passive NO_x_ monitoring). The underlying data in the model inputs or covariates may have also become more accurate or complete over time. For example, accurately capturing NO_x_ emissions in the years earlier than 2000 is much more challenging (sparser traffic volume and road network data). Given the observed time trend, our findings indicate that higher NO_x_ exposure predictions (which also occurred in earlier years) are prone to higher levels of uncertainty. Other work has found that when magnitude and uncertainty of exposure are correlated, there is a notable attenuation of the exposure response curve for high exposure values (Steenland et al., 2015), but this has not yet been formally tested in this analysis.

In addition to year of prediction increasing exposure uncertainties, we found that geographic location and other spatially dependent predictors also influenced uncertainties. The comparison of covariate distributions in areas of high and low SMME indicate that measurement error is likely associated with non-freeway sources, or sources/features found in areas further away from freeways. We saw higher uncertainty in predictions located near smaller roads (FCC2 and FCC3) and lower SMME in predictions located near freeways (FCC1). Interestingly, more uncertainty was found in locations with higher heavy-duty vehicle fractions on (FCC2) roads. FCC2 roads are very similar to FCC3 roads as they are state-numbered highways with stop and go traffic, with volumes greater than FCC3 roads but less than FCC1 roads (for example, Pacific Coast Highway, also known as Route 1 is considered an FCC2 road in southern California). Although further analysis is needed, findings indicate that the exposure model does not adequately capture NO_x_ emissions from FCC2 roads, and more specifically from heavy duty vehicles on these roads. This conclusion is further supported by the large proportion of SMME observed among predictions located in Long Beach, CA, a community with the busiest port in the nation, and therefore high proportion of heavy duty vehicles. Although some of the CHS communities do not have any FCC2 roads and the majority only have one, Long Beach includes three FCC2 roads. Our findings support the importance of accounting for local NOx sources and fine scale spatial variability in exposure prediction models, especially in regions with complex NO_x_ sources and dense development.

Distance to major airport, defined as one of the top 5 busiest airports in the study region, was an important predictor of SMME for all CHS locations but was not influential in the Long Beach only analysis. Beyond light and heavy duty vehicular NO_x_ emissions on roads, our exposure model did not account for airports although they are a major source of NO_x_ emissions, not only due to increased vehicular traffic near airports, but also idling planes and jets, takeoff and landing activity, and vehicular operations within airport boundaries (Schlenker and Walker, 2015). In our spatial analysis we found elevated odds of high SMME in geographic locations near Los Angeles International Airport and San Diego International Airport. The influence of smaller airports within the region was formally tested in a sensitivity analysis in the GAM models, but smaller airports did not influence the spatial variability or magnitude of SMME risk. We suspect the smaller airports were not important predictors of SMME as our exposure prediction model spans from 1992 to 2012, and airport operations among smaller (Class 1) airports have only recently increased. Long Beach, a population dense urban area with complex NO_x_ source mixtures, houses a single local airport and a large shipping port. Therefore, there is not much variability in the distance to the centrally-located Long Beach Airport in this city-specific analysis, and airport operations were not consistent throughout this time period.

Although we found that shared additive error was larger in magnitude than SMME, we focused our analysis on SMME as other work has indicated minimal influence of shared additive error on epidemiological results in a Berkson model (Zhang et al., 2017). Shared error differs from traditional measurement error as the errors are not independent, which is common in air pollution exposure models because (1) model covariates are usually aggregated in time and space and (2) air pollution exhibits finely resolved variability through time and space.

The SUMA method classifies “within” and “between” measurement error as unshared and shared error, retrospectively. One shortcoming of the SUMA error approach is that it does not account for “within shared error”, defined as shared uncertainties for predictions made in the same or a proximal geographic location over time. SUMA methods also do not account for “between shared error” attributable to time, for example, predictions made in the same year and month will share uncertainties. Previous simulation studies determined that shared error within predictions resulted in greater bias than shared error between predictions (Hoffmann et al., 2018). We hope to elaborate on SUMA models to enable classification of within and between shared errors in future work.

In this work, we treat the 120 ensemble estimates as 120 realizations of a dosimetry system. An assumption of the dosimetry system is that the realizations are generated from a random sample of true exposures that are normally distributed. In our application, parallel ensembles are generated using a subset of prediction points and covariates, which explain the variability of the 120 ensemble exposure prediction estimates. Parallel ensembles take full advantage of independence between base learners (Kotsiantis et al., 2007). The ensembles represent a random sample of possible exposure predictions from the distribution of possible prediction models given a single set of covariates, but the weight given to each ensemble is dependent on model performance to output stage 2 output.

One limitation of our spatiotemporal error analysis is the reliance on average covariances for each prediction to identify high SMME. Covariance is a measure of deviation between two variables. We used the average of all covariance values with all other predictions to dichotomize SMME as high or low. As covariances are unstandardized, the spatiotemporal patterns observed can be an artifact of NO_x_ absolute values since high NO_x_ predictions are likely to have higher covariances. We assume that using the 80th percentile of average covariances will capture predictions with unusually and consistently high covariances with other predictions. Although this definition captured some predictions with high absolute NO_x_ concentrations, it also classified some low NO_x_ predictions were as having high SMME.

In this analysis, we selected a sample of 2500 (0.1%) exposure predictions out of 1,850,415 possible predictions. Given the manipulation of large covariance matrices, this sample number was arbitrarily chosen to accommodate computational ability and time. Given the small proportion of represented points selected in this analysis, we compared the spatial and temporal distributions of the random sample to the entire prediction population and found the sample was spatially and temporally representative (Table 1). In attempt to determine the presence of selection bias resulting from our sampling method, we further selected 10 additional random samples. Findings indicate that SUMA error magnitude was robust across samples (Table A4). We encourage future analysis of this type, to ensure samples are spatially and temporally representative of the universe of exposure predictions.

In this paper, we developed a statistical framework to quantify the different components of measurement error in NO_x_ predictions from our previously published spatiotemporal exposure model (Li et al., 2017) demonstrating that the Stram and Kopecky (2003) radiation dosimetry framework can be applied to air pollution. We also explained the spatial (geographic) and temporal variability in the odds of observing high shared, multiplicative measurement error – the component most commonly seen in air pollution investigations. Our work highlights the ability to use ensembles the in the evaluation of SUMA error and sets up a framework to evaluate potential factors that might be responsible for exposure uncertainties. Our methods can help improve the development of future exposure models by either highlighting areas in space or periods in time where more refined data or methods are needed or shedding light on potentially important inputs or predictors that might be overlooked. Further, characterization of exposure errors can be used to improve confidence in epidemiological inference (Hoffmann et al., 2018) through adjustment of confidence intervals to account for SMME (Stram and Kopecky, 2003) or attenuation of the dose response curve (Stram et al., 2015). Given the importance of this work to exposure science and environmental epidemiology, our follow up work will focus on assessing the impact of SUMA exposure error on epidemiological health estimates and methods for adjusting them accordingly.

## Supplementary Material

1

## Figures and Tables

**Table 1:** Comparison of the distribution of estimated NO_x_ exposures^a^ and their main predictors in the full southern California Children’s Health Study Cohort E Residential (Biweekly) Timelines^b^ and in the subset of 2500 randomly sampled^c^ predictions used in the assessment of Shared Unshared Multiplicative Additive (SUMA) exposure measurement error.

N	Full CHS cohort E timelines	Random sample of 2500 predictions
	
	1,850,415	2500
	
	n (%)	n (%)
Prediction year		
1992–2000	615,454 (33.2)	826 (33.0)
2001–2004	568,177 (30.7)	749 (30.0)
2005–2012	666,784 (36.0)	925 (37.0)
Traffic density within a 300 m buffer^d^		
0–13.54	462,287 (25.0)	651 (26.0)
13.55–33.61	462,427 (25.0)	611 (24.5)
33.62–75.64	462,518 (25.0)	579 (23.1)
75.65–1235	462,591 (25.0)	659 (26.3)
Population density^e^		
0–2700	462,606 (25.0)	657 (26.2)
2701–5234	461,887 (25.0)	571 (22.8)
5235–9049	463,340 (25.0)	642 (25.6)
9050–78,668	462,582 (25.0)	630 (25.2)
Mean elevation within a 300 m buffer		
−36.6–56.5	462,790 (25.0)	648 (25.9)
56.6–253.3	462,038 (25.0)	598 (23.9)
253.4–365.4	462,892 (25.0)	633 (25.9)
365.5–2231.8	462,695 (25.0)	621 (24.8)
Distance to major roadways^f^ (meters)		
0–150	113,133 (6.1)	163 (6.5)
151–300	147,851 (7.9)	210 (8.4)
> 300	1,589,431 (85.8)	2127 (85.0)
CALINE4^g^ freeway NO_x_ (ppb)		
0–3.30	462,837 (25.0)	629 (25.1)
3.31–8.87	462,175 (25.0)	590 (23.6)
8.88–18.55	462,453 (25.0)	626 (25.0)
18.56–455	462,950 (25.0)	655 (26.2)
CALINE4^g^ non-freeway NOx (ppb)		
0–2.43	461,744 (25.0)	656 (26.2)
2.44–4.75	462,269 (25.0)	587 (23.4)
4.76–8.10	463,607 (25.0)	624 (25.0)
8.11–92.39	462,795 (25.0)	633 (25.3)
Spatiotemporal NO_x_ predictions^h^ (ppb)		
2.10–20.62	462,406 (25.0)	635 (25.4)
20.63–31.60	462,523 (25.0)	632 (25.3)
31.61–48.40	462,800 (25.0)	589 (23.6)
48.41–277.00	462,689 (25.0)	644 (25.8)

a Each prediction is for a biweekly period at a residential location from the reconstructed CHS lifetime residential history.

b Exposure prediction characteristics for all 5106 southern California Children's Health Study (CHS) cohort E participants.

c Geographic characteristics summarized for sample 1 of 10.

d Traffic Density calculated using distance decayed annual average daily traffic (AADT) volume from major roads (freeways/highways and major surface streets) within a 300 m circular buffer.

e Population density calculated within 300 m buffers based on US census block group populations from the 1990, 2000, 2010 linearly interpolated or extrapolated for 1992–2012.

f Distance to freeways/highways (FCC1 road classification).

g CALINE4 is line source dispersion model using quarterly average daily traffic volumes (Benson, 1984).

h Spatiotemporal Stage 2 NO_x_ predictions (Li et al., 2017).

**Table 2:** Distribution of between and within prediction variance parameters used to determine Shared Unshared Multiplicative Additive (SUMA) measurement error components.

Parameters	Min	Max	Mean (standard deviation)	Median	Reference
Covariance	−69	513	0.20 (3)	0	*C_ij_*, Eq. (2)
Product of means	9	43,192	1453 (1560)	967	*Z_i_Z_j_* Eq. (2)
Variance	0.02	649	11 (34)	4	*V_i_*, Eq. (3)
Square of mean	3449	43,510	2133 (3450)	975	*Z_i_*^2^, Eq. (3)

**Table 3:** Shared and Unshared, Multiplicative and Additive (SUMA) exposure measurement error components in the spatiotemporal NO_x_ predictions for the southern California Children's Health Study Cohort lifetime residential histories.

Error type	Value	Standard error	p-Value	Reference
Shared additive ($\sigma_{\mathrm{SA}}^{2}$)	−0.25166	0.00209	< 0.0001	Eq. (2), Fig. 2 (intercept)
Shared multiplicative ($\sigma_{\mathrm{SM}}^{2}$)	0.00029	0.0000009	< 0.0001	Eq. (2), Fig. 2 (slope)
Unshared additive ($\sigma_{A}^{2}$)	−5.39406	0.48467	< 0.0001	Eq. (3)
Unshared multiplicative ($\sigma_{M}^{2}$)	0.00751			Eq. (3)

**Table 4:** Time-stratified (calendar year tertiles) analysis of Shared Multiplicative Measurement Error (SMME) in the spatiotemporal NO_x_ predictions for the southern California Children's Health Study Cohort lifetime residential histories.

Time period^a^	1992–2000	2001–2004	2005–2012
Shared multiplicative error ($\sigma_{\mathrm{SM}}^{2}$) component^b^	0.0003627^c^	0.0001549^c^	0.0001496^c^
Min covariance	−89	−31	−20
Max covariance	757	240	182
Median covariance	0.7	0.5	0.4
Min product mean	40	19	10
Max product mean	52,691	24,540	15,215
Median product mean	1563	1100	589

a A random subset of 2500 predictions were sampled for each time period stratum.

b Shared multiplicative error component determined by the slope of the regression of the covariance on product means between predictions using 120 ensembles.

c p-Value < 0.0001.

**Table 5:** Spatial and temporal predictors of the odds of high Shared Multiplicative Exposure Measurement Error (SMME)^a^ in spatiotemporal NO_x_ predictions using a random subset^b^ of the southern California Children's Health Study.

	Odds ratio	95% confidence interval	p-Value
CALINE4^c^ non-freeway NO_x_	1.06	(1.04, 1.08)	< 0.0001
Population density^d^	1.03	(1.01, 1.04)	< 0.0001
Traffic density within a 300 m buffer^e^	1.11	(1.09, 1.14)	< 0.0001
Distance to major airport (km)^f^			
0–15	1.16	(1.10, 1.23)	0.0001
> 15	1.00	–	–
Time period			
1992–2000	1.00	–	–
2001–2004	0.97	(0.93, 1.00)	0.1777
2005–2012	0.90	(0.87, 0.94)	< 0.0001

a Shared multiplicative error determined as the top 80th percentile of average covariance distribution at each unique location.

b Random subset of 2500 predictions sampled.

c CALINE4 is line source dispersion model using quarterly average daily traffic volumes (Benson, 1984). Odds Ratios given for an interquartile range increase (5.89 ppb).

d Population density calculated within a 300 m buffers based on US Census block group populations from the 1990, 2000, 2010 linearly interpolated or extrapolated for 1992–2012. Odds Ratios given for an interquartile range increase (664.4 people per 300 m buffer).

e Traffic Density calculated using distance decayed annual average daily traffic (AADT) volume from major roads (freeways/highways and major surface streets) within a 300 m buffer. Odds Ratios given for an interquartile range increase 60.3 AADT per 300 m buffer.

f Distance to major (largest 5 in study area) class 1 airports in meters.

**Table 6:** Geographic characteristics of spatiotemporal NO_x_ predictions with high and low Shared Multiplicative Exposure Measurement Error (SMME) from a random sample of 2500 predictions from the city of Long Beach, California.

	Low SMME (Covariance < 80th percentile)	High SMME (Covariance ≥ 80th percentile)	p-Value ^j^	95% CI of difference (Low-high)
			
	Mean (sd)	Mean (sd)		(95% CI)
NO_x_ measures (ppb)				
Exposure model stage 2 NO_x_ output^a^	55.22 (33.54)	79.36 (39.38)	< 0.001	−24.86, −24.61
Ambient NO_x_^b^	54.81 (28.14)	77.15 (35.35)	< 0.001	−22.03, −22.65
CALINE4^c^ freeway NO_x_	29.46 (18.00)	26.61 (17.01)	< 0.001	2.79, 2.90
CALINE4^c^ non-freeway NO_x_	16.90 (13.9)	25.50 (12.7)	< 0.001	−3.63, −3.58
Traffic measures				
Traffic density^d^ within a 300 m buffer	117.84 (74.01)	126.97 (62.72)	< 0.001	−9.34, −8.92
Distance^e^ to freeways (FCC1) m	1318.81 (850.88)	1589.6 (833.4)	< 0.001	−274.17, −268.72
Distance^e^ to arterial roads (FCC2) m	3139.12 (1991.44)	2593 (2036.70)	< 0.001	539.07, 552.29
Distance^e^ to collector/distributor roads (FCC3) m	205.76 (133.77)	181.48 (124.62)	< 0.001	23.20, 24.03
Distance^e^ to local roads (FCC4) m	26.77 (13.9)	27.38 (14.62)	< 0.001	−0.656, −0.561
Heavy duty vehicle fraction FCC1^f^	0.120 (0.05)	0.125 (0.05)	< 0.001	−0.0055, −0.0056
Heavy duty vehicle fraction FCC2^f^	0.030 (0.05)	0.050 (0.06)	< 0.001	−0.0114, −0.01110
Average annual daily traffic FCC1^g^	192,745.0 (61,375.6)	185,859 (59,631.8)	< 0.001	6690.67, 7081.67
Average annual daily traffic FCC2^g^	37,635.2 (6221.5)	37,133 (5187.9)	< 0.001	483.58, 518.86
Average annual daily traffic FCC3^g^	26,127 (7253.1)	24,773 (8221.5)	< 0.001	1327.55, 1379.93
Average annual daily traffic FCC3^g^	4974 (353.9)	4866 (376.8)	< 0.001	106.69, 109.09
Meteorology				
Minimum temperature	13.57 (3.40)	11.20 (3.4)	< 0.001	1.28, 1.30
Wind speed	2.19 (0.39)	2.20 (0.41)	< 0.001	−0.017, −0.015
Other				
Elevation^h^	15.1 (3.7)	15.2 (4.2)	0.321	−0.019, 0.007
Distance^e^ to shoreline	6880.6 9 (3282.36)	5793.5 (3282.36)	< 0.001	1076.54, 1097.69
Population density^i^	14,899 (4990)	18,338 (4191)	< 0.001	−3453.23, −3424.78

a Average of 120 ensembles from Stage 2 of the spatiotemporal NO_x_ exposure model.

b Ambient NO_x_ measured at the EPA air quality monitoring stations.

c CALINE4 is a line source dispersion model using quarterly average daily traffic volumes (Benson, 1984).

d Traffic density calculated using distance decayed annual average daily traffic (AADT) volume from major roads (freeways/highways and major surface streets) within a 300 and 500 m circular buffer.

e Distances calculated in meters.

f Fraction of heavy duty vehicles by road class within 300 m buffer.

g Average annual average daily traffic at location (point estimate).

h Mean elevation in a 300 m buffer.

i Population density calculated within 300 m buffers based on US Census block group populations from the 1990, 2000, 2010 linearly interpolated or extrapolated for 1992–2012.

j Welch non-parametric two sided *t*-test.

**Table 7:** Distribution of NO_x_ predictions with low or high shared Multiplicative Exposure Measurement Error (SMME) across season or time period drawn from a random sample of 2500 predictions from the city of Long Beach, California.

	Low SMME (Covariance < 80th percentile) n^a^ (%)	High SMME (Covariance ≥ 80th percentile) n^a^ (%)	p-Value^b^
Season^c^			
Spring	542 (27.5)	76 (15.0)	–
Winter	403 (20.4)	193 (39.2)	< 0.001
Summer	568 (28.9)	47 (9.5)	< 0.001
Fall	454 (23.1)	176 (35.8)	< 0.001
Time period			
1992–2000	576 (29.3)	214 (43.4)	–
2001–2004	579 (29.5)	139 (28.2)	< 0.001
2005–2012	812 (41.3)	139 (28.2)	< 0.001

a Total sample n = 2459 after accounting for repeat predictions within sample.

b Welch non-parametric two sided *t*-test.

c Seasons defined as winter (December through February), spring (March through May), summer (June through August), fall (September through November).
