# Supplementary material for: Exposure measurement error in air pollution studies: A framework for assessing shared, multiplicative measurement error in ensemble learning estimates of nitrogen oxides
Source: Environ Int. Author manuscript; Available in PMC 2020 Apr 1. (PMC6499078; doi:10.1016/j.envint.2018.12.025)
Supplement: 1 [file NIHMS1520458-supplement-1.docx]

**Supplemental Material**

**Assessment of Shared and Unshared Exposure Measurement Error in Ensemble Learning Estimates of Nitrogen Oxides and Its Implications on Epidemiological Findings in Air Pollution Studies**

Mariam S. Girguis^a^, Lianfa Li^a^, Fred Lurmann^b^, Jun Wu ^c^, Robert Urman^a^, Edward Rappaport^a^, Carrie Breton^a^, Frank Gilliland^a^, Daniel Stram^d^, Rima Habre^a^

^a^Division of Environmental Health, Department of Preventive Medicine, University of Southern California, Los Angeles, CA, ^b^Sonoma Technology, Inc, Petaluma, CA, ^c^Department of Public Health, College of Health Sciences, University of California, Irvine, CA, ^d^Division of Biostatistics, Department of Preventive Medicine, University of Southern California, Los Angeles, CA

*Corresponding Author*

Mariam Girguis, PhD, Division of Environmental Health, University of Southern California Keck School of Medicine, SSB 230 2001 N. Soto Street, Suite 102, Los Angeles, CA 90089, Phone +1 (323) 442-2795, E-mail [mgirguis@usc.edu](mailto:mgirguis@usc.edu)

**Contents**

Table A1: **Distribution of southern California Children’s Health Study (CHS) spatiotemporal NO_x_ Predictions with High Shared Multiplicative Exposure Measurement Error (SMME) by City……………..2**

Table A2: **Predictors Considered to Model the Geographic Variability of Shared Multiplicative Measurement Error (SMME)……………………………………………………………………………………………………………..3**

Table A3: **Spatial and Temporal Predictors of Shared Multiplicative Exposure Measurement Error (SMME)^a^ in Spatiotemporal NO_x_ Exposure Prediction Models Using a Random Subset^b^ of the Long Beach predictions…………………………………………………………………………………..…………………………………………4**

Table A4: **Shared Multiplicative Exposure Measurement Error (SMME) Values of Spatiotemporal NO_x_ Exposure Prediction models for 10 Random Samples of the southern California Children’s Health Study(CHS) Cohort…………………………………………………………………………………………………………………………….5**

# Table A1: **Distribution of southern California Children’s Health Study (CHS) spatiotemporal NO_x_ Predictions with High Shared Multiplicative Exposure Measurement Error (SMME) by City.**

| City | Proportion of Sample by City (%) | Within City Proportion of Sample with High SMME (Covariance ≥ 80^th^ Percentile,^a^ %) |
| --- | --- | --- |
| Long Beach | 5.89 | 23.15 |
| San Bernardino | 5.00 | 9.47 |
| Riverside | 11.73 | 8.42 |
| Anaheim | 5.46 | 7.71 |
| Santa Barbara | 8.73 | 6.66 |
| San Dimas | 5.46 | 5.26 |
| Santa Maria | 7.13 | 4.91 |
| Glendora | 7.86 | 4.21 |
| Los Angeles | 0.67 | 3.51 |
| Upland | 7.33 | 3.16 |

^a^ High covariance values determined as the top 80^th^ percentile of average covariance distribution.

Table A2: **Predictors Considered to Model the Geographic Variability of Shared Multiplicative Measurement Error (SMME).**

| Predictor | Description |
| --- | --- |
| Regional NOx | Inverse distance weighted NO_x_ from routine ambient monitoring stations. |
| CALINE4 Freeway NOx | Line source dispersion model which includes the contribution of local motor vehicle emissions to ambient mean NO_x_ concentration from emissions on freeways (FCC1 roads). |
| CALINE 4 Non-Freeway NOx | Line source dispersion model which includes the contribution of local motor vehicle emissions to ambient mean NO_x_ concentration from emissions on roadways (FCC2-FCC4 roads). |
| Population Density | Block group population in 300 m buffers based on the 1990, 2000, and 2010 census block data linearly interpolated or extrapolated annual population density for 1992−2013. |
| Traffic Density (300m) | Average traffic density within 300m radius buffer scaled by Southern California Air Basin (SoCAB) fleet NO_x_ emission scaling factor (SoCAB fleet NO_x_ emissions scaling factor (=1 in 2002)). |
| Traffic Density (5000 m) | Average traffic density 5000m radius buffer scaled by SoCAB fleet NO_x_ emission scaling factor (SoCAB fleet NO_x_ emissions scaling factor (=1 in 2002)). |
| Temperature | Minimum monthly air temperature (grided 4km) |
| Wind Speed | Average monthly wind speed (meters/ second; grided 4km). |
| Distance to Shore | Calculated shortest distance to the Pacific Ocean (meters). |
| Distance to FCC1:4 | Calculated the shortest distance to the centerline of the nearest FCC1 through FCC4 classified roadway. |
| Elevation | High accuracy elevation at a 30 m resolution. |
| Heavy Duty Vehicle Fraction FCC1:2 | Proportion of heavy duty vehicles on FCC1 and FCC2 roads. |
| Average Annual Daily Traffic FCC1:4 | Annual average daily traffic counts on roads classified as FCC1 through FCC4. |
| Distance to Airports (Major) | Calculated shortest distance to nearest major airport. Major airport defined as top 5 busiest class 1 airports in study region. |
| Distance to Airports (minor) | Calculated shortest distance to nearest minor class 1 airport. |
| Meteorological Zones | Thiessen polygons across southern California based on meteorological station locations. |
| Zip Code | 5 digit zip codes |

Table A3: **Spatial and Temporal Predictors of Shared Multiplicative Exposure Measurement Error (SMME)^a^ in Spatiotemporal NO_x_ Exposure Prediction Models Using a Random Subset^b^ of the Long Beach predictions.**

|  | Odds Ratio | 95% Confidence Interval | p-value |
| --- | --- | --- | --- |
| CALINE4^c^ Non-Freeway NO_x_ | 1.08 | (1.03, 1.18) | 0.004 |
| Population Density^d^ | 1.33 | (1.27, 1.38) | <0.0001 |
| Traffic Density^e^ FCC2 | 0.92 | (0.87, 0.96) | 0.001 |
| Prediction Year^f^ | 0.95 | (0.89, 1.00) | 0.043 |

^a^SMME determined as the top 80^th^ percentile of average covariance distribution at each unique location.

^b^Random subset of 2,500 predictions sampled from city of Long Beach, California.

^c^CALINE4 is line source dispersion model using quarterly average daily traffic volumes. Odds Ratio given for an interquartile range increase (10.49 ppb).

^d^Population density calculated within 300 meter buffers based on US Census block group populations from the 1990, 2000, 2010 linearly interpolated or extrapolated for 1992−2012. Odds Ratio given for an interquartile range increase (1207.15 people per 300 meter buffer).

^e^ Traffic Density calculated using distance decayed annual average daily traffic (AADT) volume from FCC2 roads within a 300 meter buffer. Odds Ratio given for an interquartile range increase 18363.1 AADT per 300 meter buffer).

^f^ Year of spatiotemporal NO_X_ exposure prediction (used as a continuous variable). Odds Ratio given for an interquartile range increase (6 years).

Table A4: **Shared Multiplicative Exposure Measurement Error (SMME) Values of Spatiotemporal NO_x_ Exposure Prediction models for 10 Random Samples of the southern California Children’s Health Study(CHS) Cohort**

| Sample | N | Shared Multiplicative Error (σ^2^_SM_) Value | P-value |
| --- | --- | --- | --- |
| Sample 1 | 2,500 | 0.000213 | <0.0001 |
| Sample 2 | 2,500 | 0.000195 | <0.0001 |
| Sample 3 | 2,500 | 0.000217 | <0.0001 |
| Sample 4 | 2,500 | 0.000198 | <0.0001 |
| Sample 5 | 2,500 | 0.000226 | <0.0001 |
| Sample 6 | 2,500 | 0.000187 | <0.0001 |
| Sample 7 | 2,500 | 0.000341 | <0.0001 |
| Sample 8 | 2,500 | 0.000243 | <0.0001 |
| Sample 9 | 2,500 | 0.000224 | <0.0001 |
| Sample 10 | 2,500 | 0.000224 | <0.0001 |
